# Draft Genome Sequences of *Pectobacterium carotovorum* subsp. *actinidiae* ICMP 19971 and ICMP 19972, Two Strains Isolated from *Actinidia chinensis* with Symptoms of Summer Canker in South Korea

**DOI:** 10.1128/genomeA.00104-17

**Published:** 2017-04-06

**Authors:** Sandra B. Visnovsky, Preetinanda Panda, Robert Taylor, Andrew R. Pitman

**Affiliations:** a The New Zealand Institute for Plant & Food Research Limited, Christchurch, New Zealand; b Plant Health and Environment Laboratory, Ministry for Primary Industries, Auckland, New Zealand; c Bio-Protection Research Centre, Lincoln University, Lincoln, New Zealand; d Science Solutions for Better Border Biosecurity‡; e Scion, Rotorua, New Zealand

## Abstract

*Pectobacterium carotovorum* subsp. *actinidiae* is the causal agent of summer canker in kiwifruit plants in South Korea. We report here the draft genome sequences of two *P. carotovorum* subsp. *actinidiae* strains, ICMP 19971 and ICMP 19972, which were originally isolated from *Actinidia chinensis* with symptoms of summer canker. These genome sequences will aid in the identification of genetic traits associated with their unusual capacity to cause canker and help understanding of the threat these exotic enterobacteria pose to the New Zealand kiwifruit industry.

## GENOME ANNOUNCEMENT

*Pectobacterium carotovorum* subsp. *actinidiae* has been reported as the causal agent of canker-like symptoms of kiwifruit species *Actinidia chinensis* in South Korea (1). Canker symptoms caused by *P. carotovorum* subsp. *actinidiae* are similar to those presented by kiwifruit infected with *Pseudomonas syringae* pv.* *actinidiae biovar 3, a pandemic lineage that has had a deleterious effect on kiwifruit production worldwide (2). However, the canker caused by *P. carotovorum* subsp. *actinidiae* seems to appear only under conditions of high humidity in the summer season, with the pathogen requiring higher temperatures (~30 to 32°C) to cause disease and not producing the leaf spots characteristic of *P. syringae* pv.* *actinidiae (1). We announce here the draft genome sequences of *P. carotovorum* subsp. *actinidiae* ICMP 19971 (OHJ3) and ICMP 19972 (SYJ3), isolated from yellow kiwifruit *Actinidia chinensis* cv. Hort 16A cane cankers extruding red rust ooze in South Korea (1), which were obtained from the International Collection of Microorganisms from Plants (ICMP) (http://www.landcareresearch.co.nz/resources/collections/icmp).

A comparison of the newly acquired *P. carotovorum* subsp. *actinidiae* genomes with the genomes of *P. carotovorum* subsp. *actinidiae* ICMP 19970 (KKH3) (accession no. NZ_JRMH00000000) and other pectobacteria, such as *P. carotovorum* subsp. *brasiliensis*, *P. carotovorum* subsp. *carotovorum*, *Pectobacterium atrosepticum*, and *Pectobacterium wasabiae*, will enable the taxonomic status of *P. carotovorum* subsp. *actinidiae* to be verified. Comparative analysis will also permit the identification of genetic traits associated with the capacity of *P. carotovorum* subsp. *actinidiae* to cause canker symptoms in kiwifruit plants, which could subsequently be used for risk-based diagnostics and risk assessments of similar exotic organisms at the New Zealand border.

Pure cultures of *P. carotovorum* subsp. *actinidiae* strains ICMP 19971 and ICMP 19972 were incubated overnight in lysogeny broth medium at 28°C. Genomic DNA was isolated using a DNeasy blood and tissue kit (Qiagen). DNA samples of good quality were sequenced to generate 100-bp Illumina paired-end reads using an Illumina HiSeq 4000 system (Beijing Genomics Institute, Hong Kong). The quality of the sequence reads was checked using FastQC (Babraham Bioinformatics, United Kingdom), and low-quality reads (score of <Q30) were trimmed using Fastq-Mcf (3). The remaining sequence reads were assembled into contigs using SOAPdenovo version 2.04 (4), and the contigs were aligned with the reference *P. carotovorum* subsp. *actinidiae* genome with accession no. ALIU00000000 using the SOAPaligner version 2.21 (5, 6) to generate a draft genome sequence for both ICMP 19971 and ICMP 19972. Coding sequences for each isolate were annotated using the Prokaryotic Genome Annotation Pipeline (PGAP) (http://www.ncbi.nlm.nih.gov/genome/annotation_prok/).

The estimated draft genome sizes for ICMP 19971 and ICMP 19972 were 4,891,911 and 4,886,946 bp, respectively. They comprised 50 and 49 scaffolds, respectively, with both genomes having a 51.53% G+C content. The PGAP annotations for each genome predicted 4,171 coding sequences for ICMP 19971 and 4,201 coding sequences for ICMP 19972. A number of loci were identified as common to the two *P. carotovorum* subsp. *actinidiae* strains and other bacteria that cause diseases of woody plants, suggesting they may be required for pathogenicity of *P. carotovorum* subsp. *actinidiae* on kiwifruit. A detailed assessment of the genetic traits shared by ICMP 19971, ICMP 19972, and the previously sequenced *P. carotovorum* subsp. *actinidiae* strain ICMP 19970 will follow.

### Accession number(s).

The draft genome sequences of ICMP 19971 and ICMP 19972 are available in the GenBank database under accession numbers MPUI00000000 and MPUJ00000000, respectively. The versions described in this paper are the first versions.
